# Unlocking unexplained characteristics of MgO: a review by RILEM TC 311-MBC

**DOI:** 10.1617/s11527-026-03231-0

**Published:** 2026-09-16

**Authors:** Hoang Nguyen, Charlotte Dewitte, Fei Jin, Yiming Peng, Sanket Rawat, Tingting Zhang, Nirrupama K. Ilango, Dan Meng, Tangei Mi, Matthias Müller, Dung Nguyen, Daman Panesar, Navid Ranjbar, Shaoqin Ruan, Vineet Shah, Runxiao Zhang, Zeyu Zhou, Cise Unluer

**Affiliations:** 1 Fibre and Particle Engineering Research Unit, University of Oulu, Pentti Kaiterankatu 1, 90014 Oulu, Finland; 2 Empa, Swiss Federal Laboratories for Materials Science and Technology, Überlandstrasse 129, 8600 Dübendorf, Switzerland; 3 School of Engineering, Cardiff University, Cardiff, CF24 3AA UK; 4 Department of Civil Engineering and Management, University of Manchester, Manchester, M13 9PL UK; 5 School of Civil and Environmental Engineering, University of Technology Sydney (UTS), Sydney, NSW 2007 Australia; 6 Faculty of Infrastructure Engineering, Dalian University of Technology, Dalian, 116023 China; 7 Department of Civil and Mechanical Engineering, Technical University of Denmark, 2800 Lyngby, Denmark; 8 School of Science and Engineering, University of Dundee, Dundee, UK; 9 Institute of Structural Engineering, Zhejiang University, Hangzhou, 310058 China; 10 F.A.Finger-Institut Für Baustoffkunde, Bauhaus-Universität Weimar, Coudraystrasse 11, 99423 Weimar, Germany; 11 Department of Chemical Engineering, Imperial College London, South Kensington, London, SW7 2AZ UK; 12 Department of Civil and Mineral Engineering, University of Toronto, Toronto, Canada; 13 College of Civil Engineering and Architecture, Zhejiang University, Hangzhou, 310058 China; 14 Robinson Research Institute, Victoria University of Wellington, Lower Hutt, New Zealand

**Keywords:** Hydration, MgO, Magnesium carbonate cement, Magnesium silicate cements, MgO production, MgO characterization, Reactivity

## Abstract

MgO-based cements hold promises as alternatives to Portland cement with radically reduced carbon footprint. MgO plays a centric role in the reaction and formation of these cements. This review is a part of RILEM TC-311 MBC where we discussed the current understanding about the behaviors of MgO in magnesium carbonate (MC) and magnesium silicate (MS) cements. In addition, we reviewed practices in characterizing MgO and available processes to produce MgO in industrial scale. There are several unexpected and unexplained phenomena which likely link to the characteristics of MgO in both MC and MS cements. This distinguishes the MgO-cements from Portland cement and eventually several practices and know-how commonly used in cement research are not applicable and need further insights. Furthermore, the selection of MgO type and characteristics seems crucial to achieve the desired properties for both MC and MS cements. We also point out research needed in this topic to pave the way for MgO-based cements to progress toward their promises.

## Introduction: brief background on MgO-based binders

The rapid pace of urbanization across diverse regions and the burgeoning demand for infrastructure development have led to a steady annual increase in the production of concrete. In 2019, the cement industry generated 2.4 Gt of CO_2_, contributing 26% of industrial emissions worldwide [1]. The production of Portland cement (PC) is associated with notable greenhouse gas emissions, primarily carbon dioxide (CO_2_), attributable to the decomposition of limestone (CaCO_3_) into CaO and CO_2_ during calcination, along with emissions from fossil fuel combustion necessary for this process. Due to the mass use of PC, CO_2_ emissions related to its production account for 4–8% of global anthropogenic CO_2_ emissions [2, 3]. Since 2018, the carbon intensity of cement production has stayed nearly constant at slightly below 0.6 t CO_2_ per ton of cement [4], subject to variations in cement composition, manufacturing techniques, fuel types, and heat recovery [5].

To address these environmental challenges associated with PC, several alternative cementitious binders are increasingly being explored [6, 7]. Some of the prospective alternatives have been well developed including geopolymer/alkali-activated materials [8–11], aluminate cement [12, 13], and limestone calcined clay cements [14, 15] while magnesia (MgO)-based cement [16–18] are garnering growing attention due to several of their characteristics aligning with the concept of carbon neutrality or negativity. Reactive MgO is commonly derived from the calcination of magnesite (MgCO_3_) under 700–1000 °C. Although the calcination temperature is significantly lower than that of PC clinker (approximately 1450 °C) [19–21], the calcination of MgCO_3_ results in a higher carbon footprint (i.e., 1.55 kg CO_2_/kg MgO produced) compared to CaCO_3_ calcination (ca. 0.786 kg CO_2_/kg CaO) [22], making the extraction of MgO from this carbonate mineral unsustainable. However, MgO can also be extracted from rejected desalination brines, or Mg–silicate minerals [16, 23], which do not involve carbon in their chemical compositions. Consequently, these distinct technological pathways and the ample reserves of magnesia resources provide conducive conditions for further advancement towards low-carbon or carbon–neutral cementitious binders.

A variety of MgO-based cements, mainly differing by the main reaction products in the system, have been developed. Magnesium oxychloride (MOC) cement [24, 25], magnesium oxysulfate (MOS) cement [26, 27], and magnesium phosphate (MP) cement [28] are based on the type of salt present in the solution. Previous studies have discussed the respective advantages, limitations, and research prospects of MOC, MOS, and MP cements [23, 29]. In contrast, recent investigations have indicated that magnesium carbonate (MC) and magnesium silicate (MS) cements hold particular promise for developing carbon-negative and highly durable binders [30, 31]. MgO plays a central role in the formation and microstructural evolution of all MgO-based cements. Here, we focus specifically on MgO role in MC and MS cements due to the increased interests in their potential, characteristics, and performance.

The hardening and strength development process of both MC and MS cements starts with the hydration of MgO to form Mg(OH)_2_. MC cement includes reactive magnesia cement [32] and MgO-hydrated magnesium carbonates (HMCs) blends [33]. Within these systems, MgO hydrates to form Mg(OH)_2_ or hydrous carbonate-containing brucite (HCB) [34, 35] in the presence of carbonate ions. Upon exposure to CO_2_ (e.g., during carbon curing), various hydrated magnesium carbonates (HMCs), including nesquehonite (MgCO_3_·3H_2_O), artinite (Mg_2_CO_3_(OH)_2_·3H_2_O), hydromagnesite (Mg_5_(CO_3_)_4_(OH)_2_·4H_2_O), and dypingite (Mg_5_(CO_3_)_4_(OH)_2_·5H_2_O) are also produced [16]. In MS cement systems, the reaction between Mg(OH)_2_ and SiO_2_ can lead to the formation of magnesium silicate hydrate (M-S-H), the main strength-providing phase [31]. Typical mix recipes for these binders include MgO and various silica sources such as silica fume [36], fly ash [37], metakaolin [38, 39] and rice husk ash [40]. The characteristics of MgO play a major role in both MC and MS cements, ultimately influencing the reaction kinetics, early properties and long-term performance of these systems.

In this paper, we provide a comprehensive review of the characteristics of MgO used in MgO-based cements, particularly MC and MS cements. This work is part of RILEM Technical Committee *Magnesia-based binders in concrete* (311-MBC), *Working Group 2–Reactivity, reaction rate, and kinetics of raw materials*. To shed light on the ongoing initiatives in this area, first, we report unexplained and unexpected behaviors frequently observed in MgO-based cements, including cracking, atypical hydration-related responses, and discuss some possible explanations and links between the MgO characteristics and these phenomena. We then point out the research needed to obtain a better understanding of these binders. In addition, the characteristics of MgO are closely interconnected with the durability of produced cements which is covered by *Working Group 3: Thermodynamic considerations for stability and durability assessments* in this Technical Committee [41]. Second, we describe the current standards, methods, and practices used to characterize MgO, including some industrial practices that remain scattered in the literature. Furthermore, the potential link between the key properties of MgO and its reactivity is discussed. Last, we focus on the large-scale production of MgO with China (i.e., the world’s largest MgO producer) as a case study, and provide recommendations for the characterization and handling of MgO in practice.

## Unexpected and unexplained results

### Cracking during curing

**Fig. 1:**
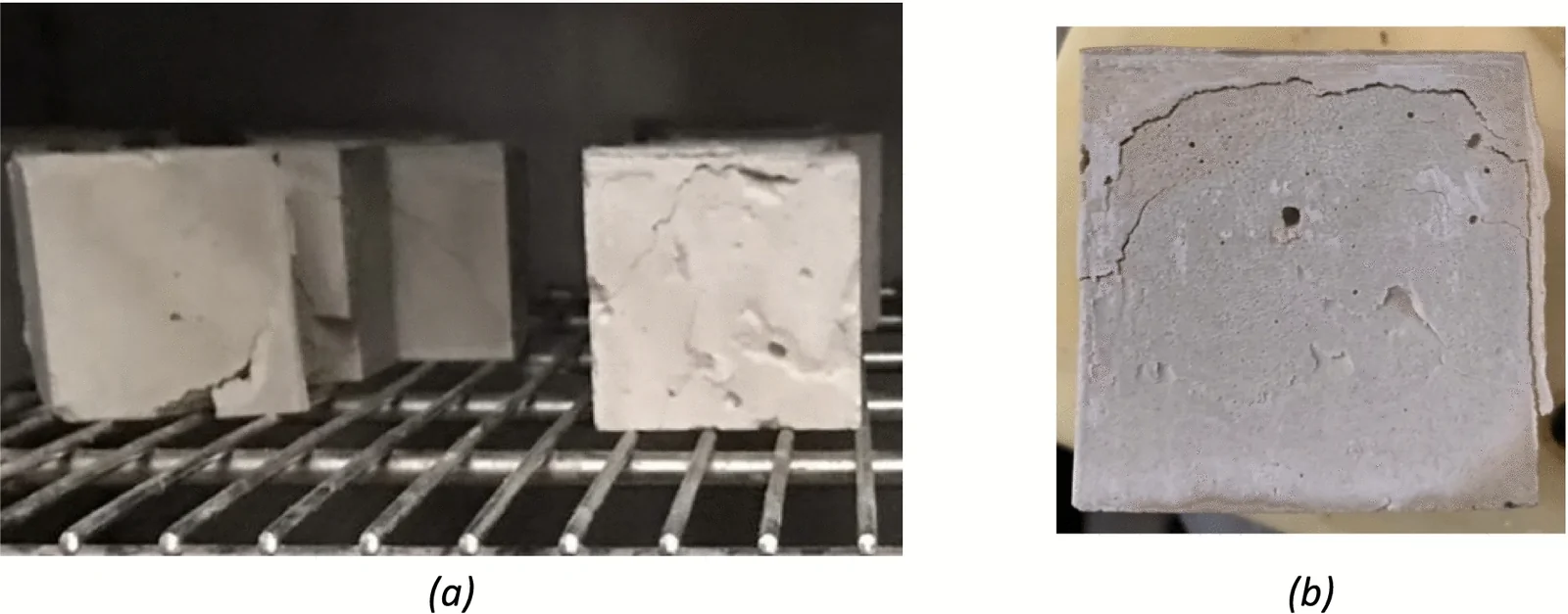
**a** Samples broken at the corner. MC concrete samples with a water/cement (w/c) ratio of 0.6, and a cement/aggregate (c/a) ratio of 0.45 and **b** Carbonation on the surface of MgO-based samples and separation from the underlying layers. MC paste sample with a w/c ratio of 0.55

During the hydration of MgO-based samples and subsequent curing, samples have been reported to develop cracks in a complex manner. In uncarbonated reactive MgO cement samples without additives, hydration can be slow due to the use of low reactivity MgO [42], resulting in samples too weak for demolding before 48 h, and leading to potential surface cracks or breakage, especially at the corners due to the adherence of the sample to the surrounding mold (Fig. 1a). Alternatively, hydration of MgO-based pastes produced with high reactivity MgO can initially cause expansion and cracks [42]. In addition, the enforced carbonation of MgO-based materials can cause rapid carbonation at the sample surface and cracking near the top of the sample [43], leading to its separation from the underlying layers. It remains unclear whether the root cause is the formation of carbonate minerals or a complex combination of MgO hydration and carbonation during the curing. Example of such cracks is shown in Fig. 1b for a sample containing 31 mass-% reactive MgO, without hydration agents, and cured under 20% CO_2_ concentration environment for 7 days.

### Cracking during storage and drying

During storage or preparation for analysis, MgO-based samples were observed to undergo drying, accompanied with cracks linked with drying shrinkage reported in pastes produced with silica or slag and MgO [44–46]. Several hypotheses have been proposed to explain these phenomena, but the exact reason remains unclear. On one side, the high-water demand of MgO often leads to high water/binder ratio (w/b > 0.5), which could weaken the paste, with cracks forming as water evaporates from the sample’s surfaces. On the other side, the formation of M-S-H observed in these pastes is thought to be responsible for the cracks [44–46]. M-S-H seems to act like swelling clay, with a high sensitivity to changes in relative humidity [44], potentially leading to the formation of cracks.

### Hydration heat inconsistencies

Across several studies involving MgO samples reported to exhibit comparable reactivity based on commonly used characterization methods (e.g. acid neutralization tests), the cumulative heat of hydration measured by calorimetry nevertheless shows substantial variation. For instance, reactive MgO calcined from brucite in the laboratory at 900 °C shows much higher cumulative heat at 72 h than the commercial reactive MgO available in the market (700 J/g [47] compared to 250 J/g of MgO [48]), indicating the influence of the chemical form of the precursor, as well as potential variations in the calcination process used to produce MgO. Mi et al. [42] reported no direct correlation between the reactivity of MgO and the cumulative heat after 3 days of hydration measured by calorimetry. The purity (i.e. the presence of CaO or unreactive part) was also reported to strongly affect the heat evolution of MgO hydration [48]. Furthermore, the raw materials (hydroxide or carbonate) also influence the crystallite size and morphology of the derived MgO where MgO derived from brucite did not show the (111) reflection and exhibited smaller crystallite size (12–15 nm) whereas carbonate-derived MgO retains the (111) with larger crystallite sizes (25–45 nm) [49].

**Fig. 2:**
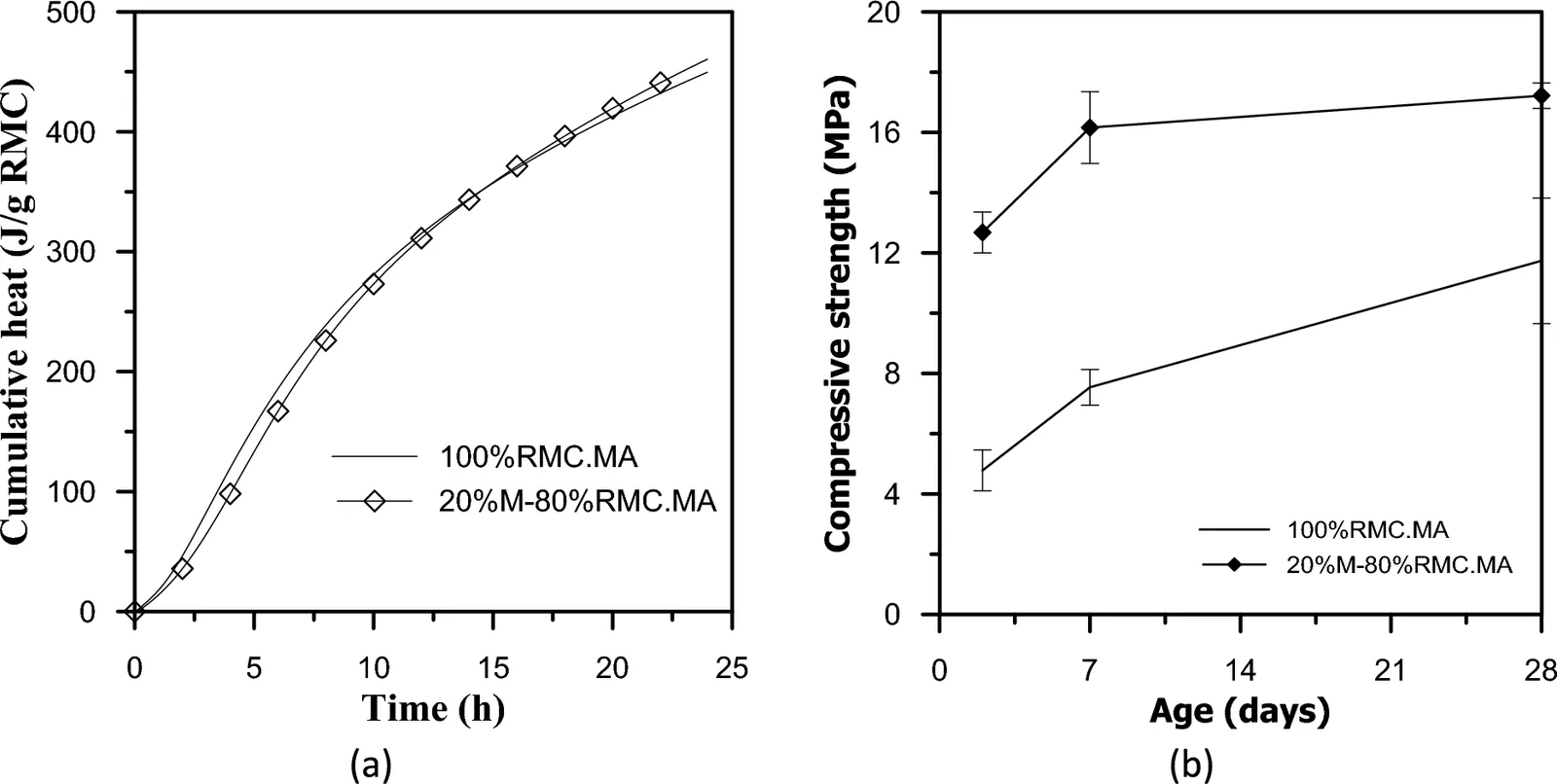
Samples reactive MgO cement with magnesium acetate (MA) and with/without magnesite (M), showing: **a** Cumulative heat per gram of reactive MgO, and **b** compressive strength (reproduced from [54])

The early age compressive strength of blended PC mixtures has been proven to have a strong linear relationship with the heat released during the hydration of cement [50–52]. However, this practical relationship is not always applicable to MgO-based cements. For example, the use of aqueous carbonate species (as NaHCO_3_ or and Na_3_H(CO_3_)_2_∙2H_2_O solutions) reduced the cumulative heat from the hydration of MgO up to 72 h, but significantly increased the 3-day compressive strength by 4–12 times [53]. In a study reported in Ref. [54], partial replacement of reactive MgO with magnesite was investigated over a range of replacement levels, both in the presence and absence of magnesium acetate (MA). For MA-containing systems, replacing 20 mass-% of reactive MgO with magnesite (sample 20–80% RMC.MA in Fig. 2a) did not result in a significant change in the cumulative heat of hydration (Fig. 2a), while a marked increase in early compressive strength (approximately 160% after 1 day) was observed (Fig. 2b). By contrast, mixes without MA showed more modest and gradual changes in both heat evolution and strength with increasing magnesite content. These results indicate that the pronounced decoupling between heat release and early strength development is most evident in the presence of MA, rather than being a general consequence of magnesite replacement alone.

The addition of 4% magnesium carbonate on MS cement showed negligible changes in the cumulative heat before 72 h compared to the pure MS cement, but again higher compressive strength at 3 d was observed [55]. The strength gain in the presence of carbonates is highly likely due to the incorporation of the carbonate/bicarbonate group into the brucite structure [34, 35]. The source of silica might also be an influential factor in this phenomenon. Sonat and Unluer [40] found that the heat release-to-strength relationship was inversed when using rice husk ash (SiO_2_ content > 75%) and micro-silica in MS pastes. The microsilica-containing sample showed the highest heat release (~ 150 J/g) in the first 3 d, but lower compressive strength (~ 6.5 MPa), whereas the sample containing rice husk ash presented lower heat release (~ 60 J/g) but higher 3 d compressive strength (~ 22 MPa). Even using two different types of rice husk ash was found to result in inverse heat release-strength relationship. Thus, unlike PC-based binders, the relationship between early hydration heat and strength development in MgO-based cements is strongly system-dependent. In MC systems, strength is largely governed by carbonation reactions, which can mask any correlation with hydration heat, whereas in MS systems a clearer relationship may be observed under certain conditions [56, 57]. Overall, early hydration heat cannot be used as a general predictor of strength across different MgO-based cement systems.

### Unclear role of carbonate phases

In some MC mixes, it remains unclear whether magnesium carbonate phases contribute to strength development. Mortars with only MgO and sand presented compressive strength up to 12.1 MPa after 3 d of moisture curing [43]. XRD and TGA data showed that only MgO and brucite were present. Traces of nesquehonite were potentially observed but might also have been present in the raw material, as the XRD of the raw MgO was not shown. Additionally, there was no broadening of the brucite reflection peaks in the XRD pattern, and no physically bound water was observed by TGA that would prove the formation of hydrous carbonate-containing brucite (HCB) [34]. However, the replacement of MgO with magnesite (MgCO_3_) by 20 mass-% in MC cement resulted in a significant strength improvement of paste samples after curing in ambient conditions from 5 to 12 MPa without any hydration agents added, and from 11 to 17 MPa when hydration agent (magnesium acetate) was used [54]. Again, neither HMCs nor HCBs were detected in the XRD patterns. In this case, acetate may have played a major role in modifying brucite’s structure as seen similarly to HCB [58].

Sodium carbonates used as additives in MS cement have been reported to improve the formation of M-S-H [59]. However, in such systems, the observed effects may not originate solely from carbonate species but could also be partially associated with the presence of alkalis. Despite this, no evidence for the formation of Mg-carbonate phases, either in crystalline or amorphous form, is usually observed in these systems [60]. A recent study, where M-S-H were wet carbonated to form nesquehonite [61] showed that no Mg-carbonates were formed [62]. These recent findings might indicate a formation of a Mg-carbonate barrier in the presence of dissolved silicon, similar to the delayed conversion of MgO to brucite in solution in the presence of dissolved silicon [63]. However, this remains to be proven, requiring further investigation and verification. Finally, in the presence of alumina in MS cement, a decrease in the compressive strength with the addition of Na_2_CO_3_ was observed and attributed presumably to the excess use of superplasticizers, but again, no specific carbonate phases could be observed in the phase assemblages [64].

### Inconclusive effects of polycarboxylate ether superplasticiser on strength development

The influence of polycarboxylate ether (PCE) superplasticizers on the strength development and fresh properties of MgO mixes remains inconclusive. The incorporation of eight types of PCE was reported to increase the 28-day compressive strength of MgO mortars by 18–136% [65]. However, scanning electron microscope (SEM) and XRD analysis revealed no significant changes in the microstructure of hardened pastes. The improved mechanical strength is likely due to enhanced particle dispersion and reduced interparticle friction, which lead to better particle packing. However, these effects were not directly measured and require further investigation. The mechanisms by which PCEs cause strength variations and their influence on MgO hydration should be systematically investigated.

MgO cement exhibited relatively high thixotropic hysteresis loop area compared with Portland cements [65–67]. The use of PCE was found to improve the storage modulus and critical shear strain of MgO paste, while reduces thixotropic behavior, shifting from the shear thinning behavior to Newtonian flow at higher shear rates [65]. Additionally, the MS cement and reactive MgO-metakaolin binders demonstrated good structural recovery after the oscillatory shear test with storage modulus recovery rates of 0.70–0.77 [57, 68]. This distinct rheological behavior can be attributed to that Mg^2+^ has strong positive charge density than Ca^2+^, resulting in strong electrostatic interaction with water molecules and consequently higher viscosity at low stress [69]. However, the correlation between thixotropic behavior and hydration mechanisms of magnesium binders remains unclear.

### Open questions

Throughout Sect. 2, we have presented experimental observations from the existing literature on MC and MS cements, where mechanisms driving these phenomena remain unclear. There is a need for better understanding of the role of MgO as the major raw material in both MC and MS cements. This includes their physical and chemical characteristics, and the relation between these properties and the macro-scale behavior of MgO-based binders. Therefore, in the remaining parts of this review, we attempt to address several important open questions that arise and provide our assessment to the current literature, laying the initial steppingstone for further insights into the performance of MgO-based cements as follows:

- How to better understand the characteristics of MgO and assess its reactivity? (Sects. 3 and 4)
- What are the current industrial practices to produce mass MgO? (Sect. 5)

## Methods for characterizing MgO

### Types of MgO

MgO is primarily manufactured via two routes: dry and wet. The dry route entails calcining magnesite, but this process has a high carbon footprint. In contrast, the wet route involves precipitation of magnesium hydroxide from seawater or magnesium-rich brines using lime/residual alkaline sources as precipitating agents, followed by low-temperature calcination to yield MgO. At the laboratory scale, MgO can also be synthesized from precipitated brucite, acquired through diverse chemical procedures, or from magnesium carbonates formed by the carbonation of magnesium-rich solutions. When derived from synthetic brucite or carbonate sources, the product is referred to as synthetic or precipitated magnesia. Additionally, MgO can also be extracted from Mg-silicates which are abundant and carbon-free. Therefore, both the wet route and Mg-silicates based extraction offer low-carbon pathways for large-scale MgO production [23].

The thermal treatment significantly influences the physical and chemical characteristics of MgO. Accordingly, MgO calcined from magnesite is often classified based on its calcination temperature, as outlined below [70, 71]:

- *Light-burned*: Calcined below 1000 °C, exhibits highest reactivity and specific surface area (SSA); primarily used in cements, as a thickener catalyst or boiler additive, in lubricating oils, and rubber compounding [72].
- *Hard-burned*: Calcined between 1000 and 1400 °C, has both lower reactivity and lower SSA. It is commonly used in animal feeds, fertilizers and pharmaceuticals. MgO naturally contained in PC also belongs to this category [73]. It is also applied in dam construction to mitigate thermal shrinkage [71].
- *Dead-burned*: Calcined at 1400–2000 °C, has very low SSA and is unreactive with crystal size ranging from 30 and 120 µm [74]. It is almost exclusively utilized in refractory applications and is known for its excellent resistance against iron oxide, alkalis, and high lime fluxing agents [70]. It is also used in magnesium phosphate cement (MPC).
- *Fused*: Calcined above fusion point of MgO (> 2800 °C, typically 12 h). It is the least reactive among the four and has very large periclase crystal (> 1000 µm). Fused magnesia exhibits superior strength, abrasion resistance, and chemical stability compared with dead-burned magnesia [74]. Approximately 85% of global MgO production comprises low-reactivity variants (dead-burned or fused) as their primary application area is refractory products [75].

MgO properties such as reactivity, pH, loss on ignition (LOI), SSA, and chemical composition vary by source and calcination method. Canterford [74] proposed that the primary properties for evaluating light-calcined magnesia include SSA, reactivity, and particle size distribution. Conversely, the evaluation of fully calcined magnesia focuses on parameters such as specific gravity and chemical composition, with particular attention to the contents of CaO, MgO, B_2_O_3_, Al_2_O_3_, Fe_2_O_3_, and SiO_2_. Here, we further elaborate on the classification and primary characterization techniques for MgO.

### Classification of MgO

This section briefly outlines the classification of MgO according to reactivity-related characteristics. Detailed discussions of the corresponding test methods, criteria, and classification boundaries are presented in the following sections (Sects. 3.3–3.7), alongside the description of the relevant characterization techniques.

The characteristics of MgO, such as reactivity, texture properties, morphology, and pH, may vary significantly depending on the source, potentially affecting its effectiveness in various applications. Jin and Al-Tabbaa [71] identified the impurities content (or pH as an indicator) and reactivity as the two most important factors to classify MgO. For characterizing reactivity, accelerated reactivity test is a widely employed method, classifying MgO into three categories (Table 1) according to the Chinese standard CBMF 19-2017 [76]. Alternatively, Chau and Li [77] proposed classification of MgO into four categories according to its suitability for specific applications (Table 2).

**Table 1:** MgO classification on the basis of reactivity via citric acid solution [76]

Class	Reactivity (seconds)*	Reactivity level
R	< 90	High
M	180–240	Moderate
S	> 240	Low

**Table 2:** MgO classification on the basis of reactivity via acetic acid solution [77]

Class	Reactivity (seconds)*	Application area
First	< 90	Suitable for research purpose
Second	90–180	Suitable for industrial application
Third	180–240	
Fourth	> 240	For special application

However, the varying grain size distribution in industrially produced MgO can lead to inaccurate reactivity assessments through the citric acid test (detailed in Sect. 3.3), where a batch of MgO with a short reaction time may expand in the long term due to the presence of low-reactive grains, and vice versa. Cao, Miao and Yan [78] thus proposed a modified neutralization method, where MgO can be divided into four categories according to the dissolution time (Table 3). Building on this categorization, low-reactivity MgO has also been reported to induce delayed expansion and cracking in hardened systems due to its slow hydration, highlighting that both high and low MgO reactivity can be problematic.

**Table 3:** Reactivity of MgO based on reaction time

Dissolution time	Reactivity
< 2 min	High reactivity (minimal expansion)
2–16 min	Medium reactivity (effective expansion agent during cement hardening)
16–90 min	Low reactivity (may cause crack post-hardening)
> 90 min	Inert and unlikely to cause expansion

For reactive grade magnesia, Jin and Al-Tabbaa [71] further recommended to assess SSA and agglomeration ratio (AR) (Table 4). However, while the primary characterization properties have been covered in this section, the selection of MgO is not solely determined by these factors as it also depends on the specific application.

**Table 4:** MgO classification on the basis of reactivity, SSA and AR [71]

Category	Reactivity (seconds)*	SSA (m^2^/gm)	AR	Feature
I–Highly reactive	< 30	> 60	< 1.5	Very quick hydration, and SSA instead of acid neutralization tests are recommended to evaluate the chemical reactivity properly
II–Medium reactive	< 300	> 10	< 3.5	Reactivity is proportional to SSA, 20–50% hydration completes in 1 day
III–Less reactive	> 300	< 10	> 3.5	Very slow hydration, special care must be taken due to risk of expansion at later stage

^*^Reactivity was measured with 0.25 M acetic acid solution

### Chemical reactivity

Reactivity critically affects MgO performance in cement due to its role in hydration and carbonation. While purity, particle size, surface area, and thermal history influence reactivity, accelerated reactivity tests are often preferred for efficiency. These fall into two main types: chemical reactivity value (reaction time) and reactivity content value (mass-based hydration). It should be noted that these two values are fundamentally different and not comparable.

#### Neutralization test—chemical reactivity value

This is the most common test for assessing the reactivity of MgO. It involves reacting a fixed quantity of MgO with an acid solution under gentle stirring and measuring the time taken to neutralize the acid. The most widely used acids are acetic acid and citric acid, though other acids like phosphoric acid and potassium dihydrogen phosphate may also be utilized [77]. In the acetic acid test, 5 g of MgO is introduced into 400 mL of 0.25 M acetic acid [70]. In the citric acid test, 1.7 g (0.042 mol) MgO is reacted with 200 mL of 0.07 mol/L citric acid at 30 ± 1 °C [79]. The time taken for phenolphthalein to change colour is recorded as the reactivity value [80].

**Fig. 3:**
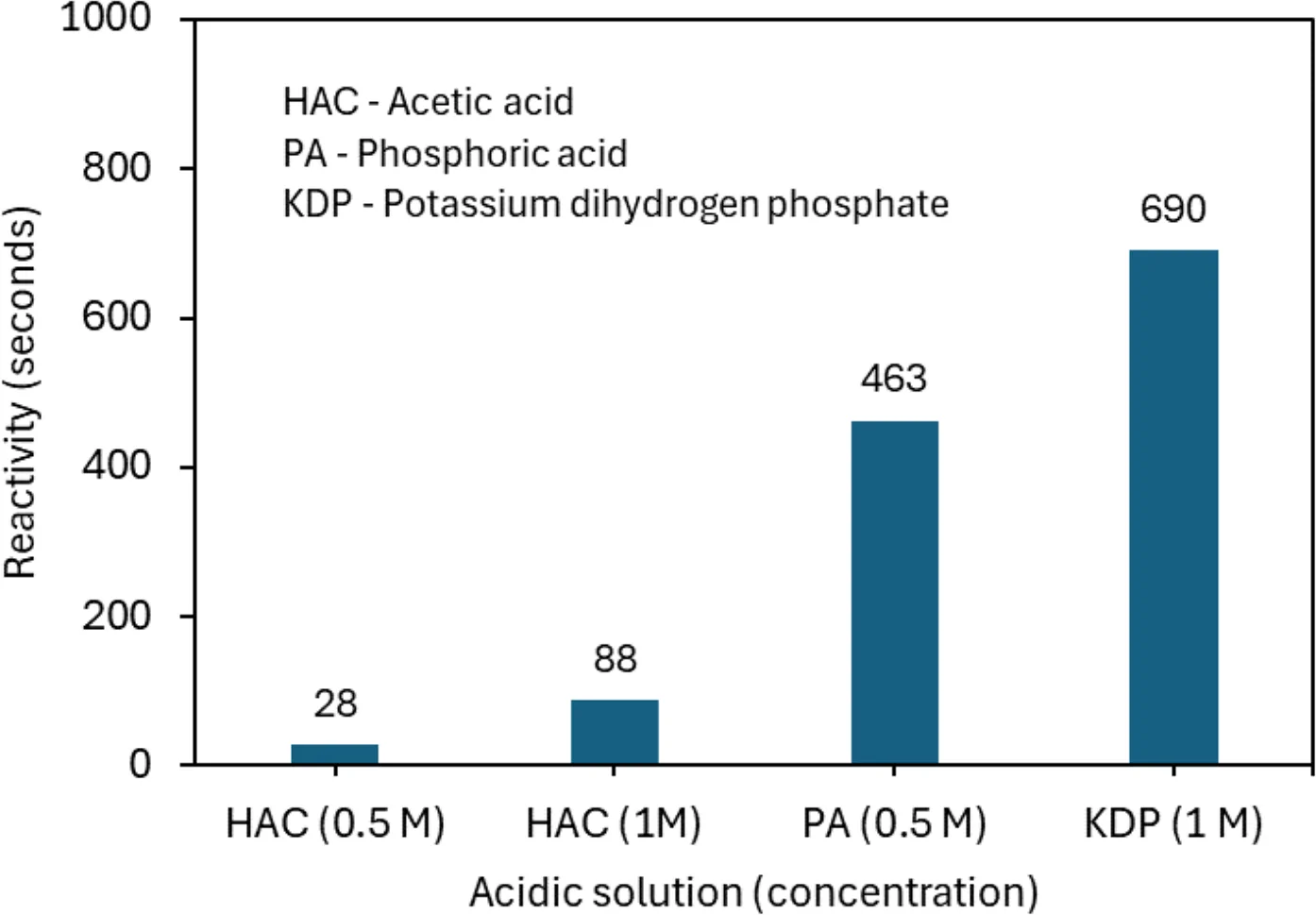
Reactivity of MgO with different types of acidic solution (reproduced from [77])

It is important to note that the solubility of major salts produced during the reaction between MgO and the acidic solution can influence the reaction rate and duration of neutralization (Fig. 3). Therefore, a weak acid, together with a water-soluble magnesium salt formed during neutralization (e.g. magnesium acetate), is generally preferred in order to avoid the precipitation of poorly soluble reaction products and to ensure a sustained neutralization process. Figure 3 recommended acetic acid use for a cost-effective and efficient estimation. [70, 76]

#### Modified neutralization method—chemical reactivity value

Cao, Miao and Yan [78] identified [78] that citric acid test can misrepresent industrial MgO due to grain size heterogeneity. To overcome this limitation, they proposed a method where 0.02 mol of MgO is reacted with 100 mL ethylic acid buffer solution (containing 1 mol/L sodium acetate and 2 mol/L ethylic acid) at 10 ± 1 °C. The reaction is stopped by decompression filtration at specified intervals (2, 16, 90 min). The residue is then rinsed with ethyl alcohol to minimize interference, dried and weighed to analyze MgO dissolution and determine reactivity.

#### WB/T 1019 (2002) test—reactivity content value

This method measures MgO reactivity based on weight gain from hydration. Typically, 2 g of MgO is mixed with 20 mL of distilled water and hydrated for 24 h at 20 ± 2 °C and 70 ± 5% relative humidity. Following hydration, the sample is dried at 105 ± 5 °C for 1 h and then heated to 150°C until constant weight. The dried sample is cooled in desiccator and weighed. The active MgO content (%) is calculated using Eq. 1.

1 $${\mathrm{W}} = \frac{{{\mathrm{W}}_{2} - {\mathrm{W}}_{1} }}{{0.45{\mathrm{W}}_{1} }} \times 100$$

where W_1_ is initial sample weight (g), and W_2_ is weight after hydration (g), and 0.45 is the reduction factor.

#### CN103115837A method—reactivity content value

This method is similar to WB/T 1019 but optimized for MgO-based expansion agents in concrete [81]. In this test, a fixed mass of MgO is first dried at 150 ℃ for 2 h and cooled in a desiccator. NaOH is separately diluted to prepare solution of pH 13 to simulate the cement medium. Subsequently, a predetermined mass of preheated MgO (denoted as W_1_) is added into 100 mL of the buffer solution and placed in a water-bath maintained at 80 ± 1 ℃. The mixture is stirred at 750–800 rev/min for approximately 5 h. The mixture is filtered, washed with absolute ethyl alcohol and the residue is dried to constant weight at 150 ℃ (denoted as W_2_). The active MgO content can then be determined using Eq. 1.

### Chemical composition

EN 14016-2:2004 (E) [82] outlines wet chemistry protocols for MgO in cement. Modern techniques such as XRF, AAS or ICP offer faster and more accurate alternatives. For XRF, samples can be prepared as finely ground pressed powder or as fused discs using lithium metaborate/lithium tetraborate flux, with the fusion method providing higher accuracy. AAS and ICP require dissolution of sample in hot acid, typically nitric, nitric/hydrochloric, or perchlorate/nitric mixtures (selected based on the solubility of silica impurities). For acid insoluble phases, lithium borate fusion technique can be employed, followed by dissolution in acid solution. It is important to note that direct magnesium measurement using AAS requires significant dilution to align its concentration within the linear range of the instrument detector, increasing the risk of dilution errors. Therefore, magnesium content is often best determined by the difference method, i.e., measuring all other impurity components of the oxide (e.g., Ca, Si, Fe, and Al), subtracting their sum from 100%, and reporting results on oxide basis.

In addition, LOI can be used to assess residual magnesium carbonate, residual magnesium hydroxide (in case of synthetic MgO) and other unaltered impurities. It is determined by the percentage weight loss after heating a fixed quantity of MgO to 1000–1150 °C. Lower LOI generally suggests higher calcination temperatures or longer durations, often correlating with lower reactivity [83].

### Specific surface area

SSA, often measured via BET nitrogen adsorption (ASTM C1274-10), reflects MgO reactivity and thermal history [84]. This method assesses monolayer gas adsorption, and pore structure can be further analyzed via the Barrett-Joynet-Halenda (BJH) method [85].

**Fig. 4:**
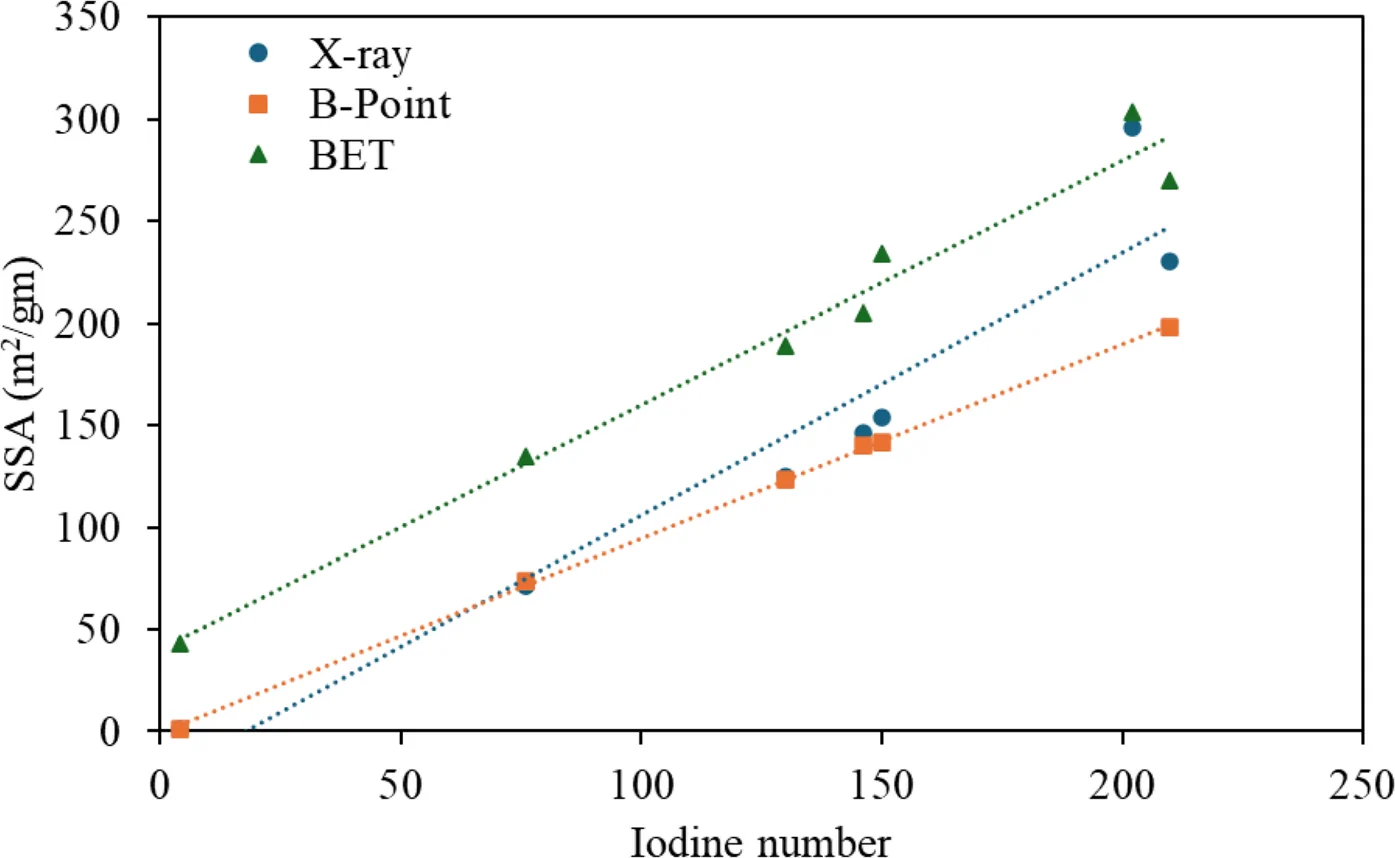
Correlation between SSA and iodine number (reproduced from [86, 87])

SSA also correlates with iodine number, a parameter traditionally used to characterize the adsorption capacity of activated carbon and commonly interpreted as a measure of the amount of iodine adsorbed per unit mass of material under specified conditions. As such, the iodine number provides an indirect indication of accessible surface area associated with fine pores [3]. Figure 4 illustrates the relationship between SSA values calculated by different methods (B-point, BET model, X-ray) and iodine number, as reported by [86, 87]. By comparing the two test values of MgO samples with SSA (B-point) from 0.73 to 198 m^2^/g, Zettlemoyer and Walker [4] reported an empirical relationship: SSA = (0.95 × iodine number).

### Particle and crystallite size and agglomeration ratio value

#### Sieving and light scattering—particle size

For coarse particles (> 150 μm), ASTM sieve analysis is appropriate. Finer particles, prone to agglomeration, require air jet sieving or laser diffraction particle size analysis, typically performed in dispersion media such as ethanol or isopropanol, to minimize particle agglomeration [2].

#### X-ray diffraction—crystallite size

Crystallite size is estimated via XRD using the Scherrer equation [88].

2 $$G_{{{\mathrm{XRD}}}} = \frac{K\lambda }{{\beta \cos \theta }}$$

where λ represents the wavelength of X-radiation (1.5405 Ǻ for Cu-Kα) [89]. θ is the Bragg angle and β broadening of the line, typically measured as the full width at half-maximum intensity of a Bragg reflection (corrected for instrument broadening) [71]. K is constant and its value depends on the method of measurement of β and shape of the particle, typically 0.89 (Scherrer) or 1.11 for spherical particles with cubic symmetry (Patterson) [90].

#### BET method—agglomeration ratio (AR)

The BET SSA value can also be utilized to estimate the primary particle size (G_BET_) using Eq. 3 [88].

3 $$G_{{{\mathrm{BET}}}} = \frac{1000F}{{S\rho }}$$

Here, ρ is the theoretical density of MgO (3.595 g/cm^3^). S is the SSA in m^2^/g and F is a particle-shape factor, typically taken as 6 for cubic particles.

It is important to note that a primary particle does not always comprise of a single crystal but often consists of several crystallites, whose average size can be denoted as ${\mathrm{G}}_{\mathrm{X}\mathrm{R}\mathrm{D}}$. These two parameters can further be used to determine AR using Eq. 4. A smaller AR would indicates that the primary particles are composed of softer agglomerations of crystallites.

4 $${\mathrm{AR}} = \frac{{G_{{{\mathrm{BET}}}} }}{{G_{{{\mathrm{XRD}}}} }}$$

### pH

Two methods are usually followed to determine the pH. In the first method, MgO powder is stored at 37 °C and 100% relative humidity for varying durations, then crushed and mixed with distilled water at a liquid-to-powder ratio of 200 mL/g. pH is measured after equilibrium is reached [91]. The second method involves mixing a fixed mass of MgO (typically 10 g) with (100 g) distilled water and taking measurement using a standard pH meter [71].

## Link between MgO properties and reactivity

The performance of MgO-based cements depends on the hydration and carbonation of MgO itself, as well as its interaction with other components in these binders. There are a vast number of different tests available to describe the various physio-chemical properties of MgO, as mentioned in Sect. 3. Among these, the chemical reactivity measured by acid neutralization tests is mostly used in the literature to indicate the quality of MgO, which is determined by its intrinsic properties such as crystal distortion and SSA. The chemical reactivity of MgO also reflects the source and calcination history of its precursors. This section collates literature data of various properties of different MgO-based binders and explores the link between these properties and MgO’s chemical reactivity.

**Fig. 5:**
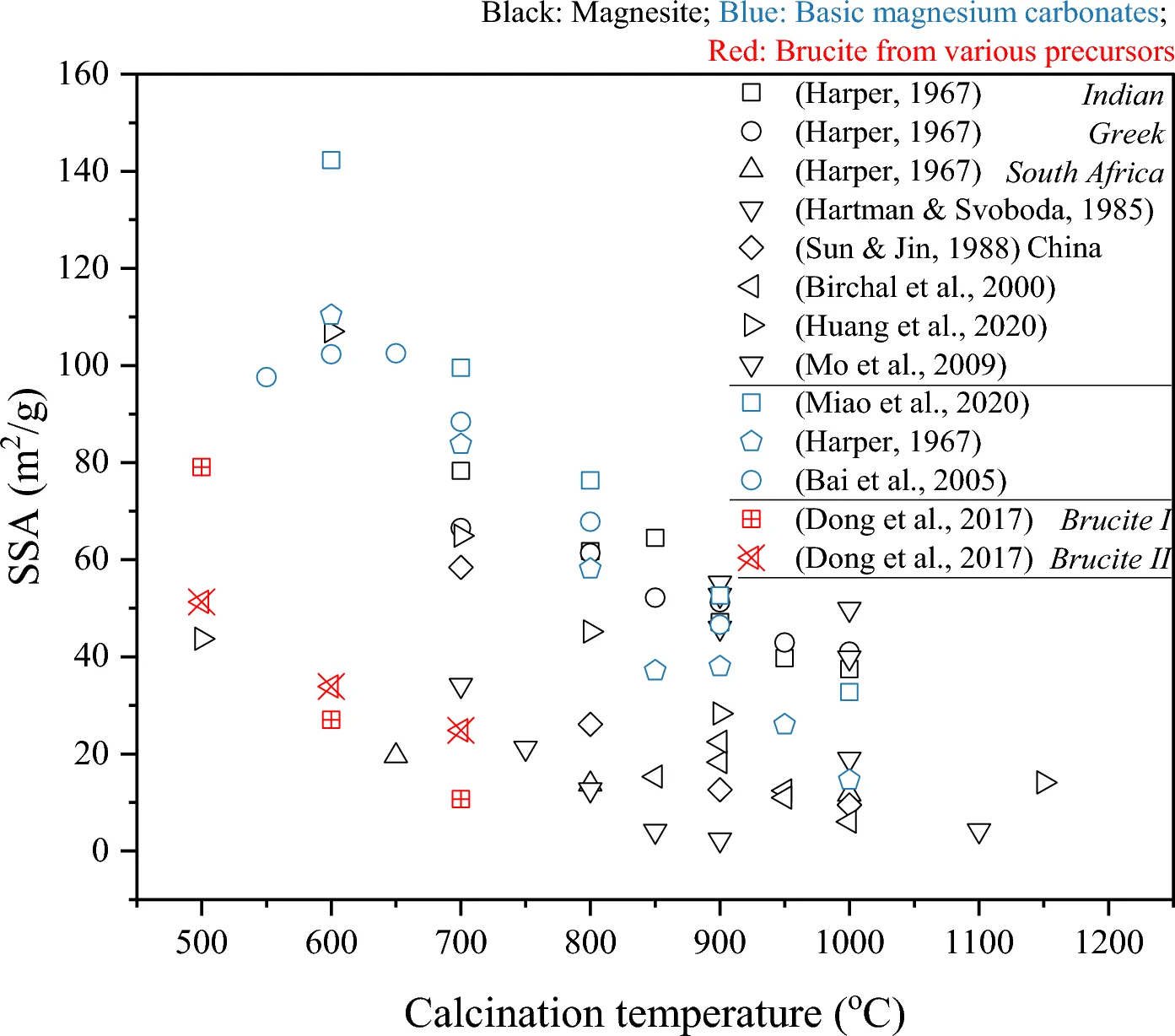
Effect of calcination temperature and precursor on the SSA of MgO samples (Data from [92–99])

**Fig. 6:**
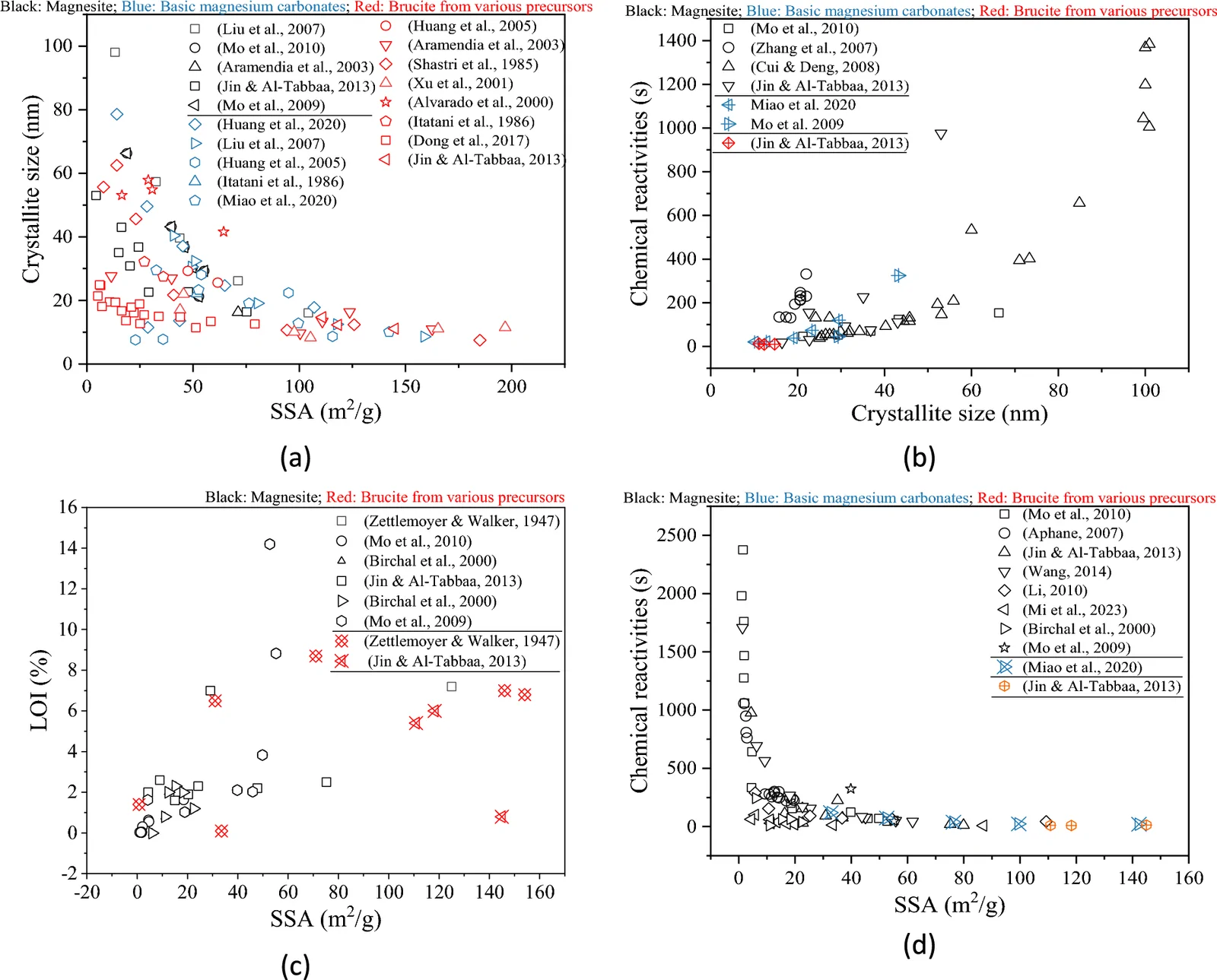
Links between **a** SSA and crystallite size, **b** crystallite size and chemical reactivity, **c** SSA and LOI, and **d** SSA and chemical reactivity (Data from [42, 71, 79, 88, 95–110])

Figure 5 shows that the precursor has a significant impact on the change of SSA during the calcination process. Therefore, solely providing information on the calcination conditions under which MgO is produced does not necessarily predict its chemical reactivity. Though SSA demonstrates a general decreasing trend with increasing calcination temperature, the varying physicochemical properties of the precursors result in differences in their thermal behavior and microstructural evolution during calcination (Fig. 6a). Similarly, chemical reactivity cannot be only reflected by the crystallite size (Fig. 6b). Figure 6c shows the correlation between SSA and LOI. No clear relationship between the two values can be observed when MgO samples produced from different routes are compared. The relationship between SSA and chemical reactivity values from different tests is plotted in Fig. 6d. Regardless of the reactivity test method and precursor used, MgO is more reactive with increasing SSA. As reactivity is expressed here as acid neutralization time (s), the “reactivity values” as such are decreasing with increasing SSA. More importantly, above a certain value of SSA (~ 10 m^2^/g), the reactivity value hardly changes with an increase of SSA. The relationship also reveals a drawback of the chemical reactivity test for the evaluation of MgO reactivity since it cannot differentiate MgO with very high reactivities. Since a reactive grade of MgO usually has an SSA of > 10 m^2^/g, it is recommended that additional methods such as SSA are used to estimate reactivity instead of solely relying on chemical reactivity measured by the acid neutralization methods.

It should also be noted that residual, incompletely calcined precursor phases, such as brucite (Mg(OH)_2_, which may also originate from the pre-hydration of MgO during transportation and storage) or magnesium carbonate, can interfere with the interpretation of the acid neutralization result. These phases could react with the test acid at markedly different rates to crystalline MgO, and their presence may bias the measured reactivity value independently of the intrinsic reactivity of the MgO phase itself. Residual carbonate and hydroxide content could be indirectly assessed via LOI (Sect. 3.4), or directly via quantitative XRD or TGA-MS or -FTIR, and should ideally be considered alongside acid neutralization data when interpreting MgO reactivity.

## Large-scale production of MgO: the case in China

China is the largest producer of MgO worldwide, with mature industrial infrastructure and technical know-how in magnesia production. Among the various production routes, magnesite calcination remains the dominant method due to the country’s abundant magnesite resources and well-established processing chains. Industrial magnesia is conventionally produced by calcining magnesite in shaft kilns, directly fired rotary kilns, fluidized-bed calciners, or suspension/flash calciners. Shaft kilns are relatively simple and thermally efficient but generally require coarse and uniformly sized feed and may suffer from non-uniform temperature and residence-time distributions. Rotary kilns provide better mixing and can accommodate a broader range of feed sizes, although heat losses and direct contact between the combustion gas and the material may increase energy demand and dilute the CO_2_-rich calcination gas. Fluidized-bed and suspension/flash systems offer rapid heat and mass transfer and more uniform calcination of fine powders, but require tighter feed preparation, more complex gas–solid separation, and stricter process control [111, 112].

However, high-grade magnesite reserves are rapidly declining due to continuous mining, posing a challenge for sustainable MgO production. Low-grade magnesite, which cannot be directly used as feedstock for high-quality refractory or cementitious materials, has attracted growing attention. To better utilize this resource, beneficiation techniques such as flotation are employed to remove impurities like silica, upgrading low-grade ore into suitable raw material for calcination [113]. Developing efficient beneficiation and calcination processes for low-grade magnesite is essential to increase MgO content and reduce impurities such as CaO and SiO_2_. In particular, the flotation purification of low-grade (MgO 35–45%) and especially ultra-low-grade (MgO < 35%) magnesite has become a key research focus in the magnesite processing field [114]. In this Section, several technologies will be discussed aiming to introduce an overview on the MgO production in China for product uniformity, fine-feed utilization, energy recovery, and the potential for CO_2_ capture.

### External heating rotary kiln calcination

**Fig. 7:**
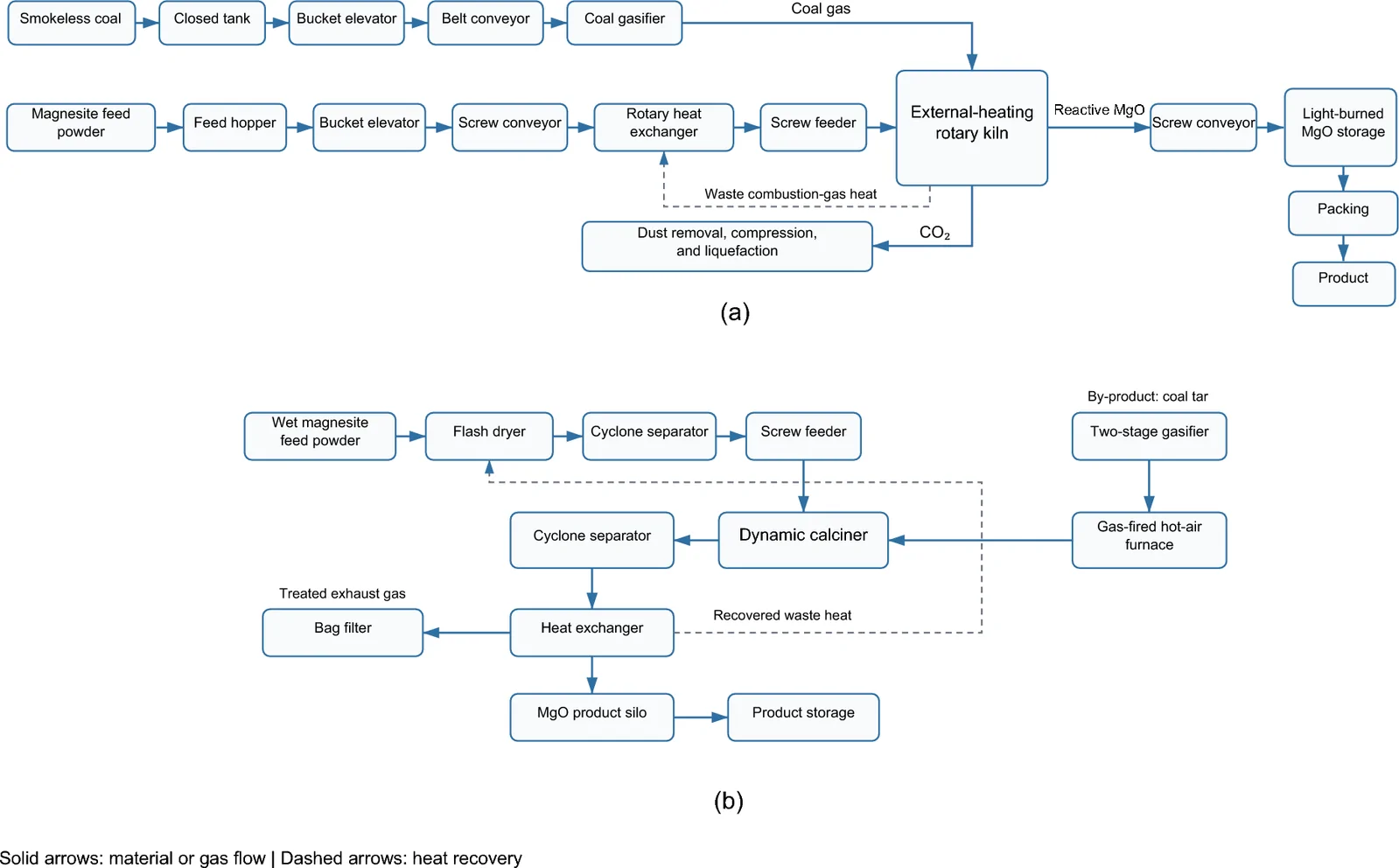
MgO production process involving **a** external heating rotary kiln calcination and **b** flash cyclone dynamic calcination

The utilization of an externally heated rotary kiln enables the production of high purity activated MgO, while facilitating CO_2_ capture, thereby mitigating environmental pollution. Incorporating the rotary kiln as the primary apparatus, an exterior combustion chamber is constructed around the rotary kiln cylinder. Gas is utilized as the fuel for heating the rotary kiln cylinder indirectly and uniformly. Concurrently, a spiral feeder facilitates uniform feeding into the rotary kiln, ensuring consistent magnesite feed distribution within the kiln. Under reduced-pressure conditions, the powder undergoes uniform heating and decomposition as the cylinder rotates, resulting in the production of activated MgO and CO_2_ gas [115]. Calcination temperature ranges from 850 to 1100 °C. In Dashiqiao City, Liaoning Province, magnesite feed has been utilized as a raw material for the production of reactive high-purity MgO, with simultaneous CO_2_ recovery achieved using an externally heated rotary kiln [116]. The production process flow chart is depicted in Fig. 7a.

### Flash cyclone dynamic calcination

Flash cyclone dynamic calcination presents significant research interest towards low energy consumption, minimal carbon emissions, and optimal efficiency in magnesite calcination processes. The calcination process utilizes either gas combustion or natural gas as the heat source. Initially, the residual heat from the calcination furnace is drawn into the drying unit under negative pressure. Raw mineral powder or moisture-laden powder is continuously fed into the drying unit. Through rapid mass and heat transfer with hot air, moisture molecules vaporize instantaneously, achieving dehydration to ≤ 1% within ~ 5 s, completing the drying phase. The material then goes to cyclone and baghouse dust collectors for gas–solid separation. Purified exhaust gases are extracted by an induced draft fan and directed into a preheater for further heating. After undergoing a multi-stage preheating process, the material is introduced into the flash cyclone dynamic calciner, where particles undergo thorough thermal decomposition reactions within the rotating upward airflow. Exposed to temperatures ranging from 950 to 1200 °C for 3–5 s, the particles release CO_2_, forming porous MgO particles. This method effectively mitigates the risk of over- or under-firing. Following separation, the powder is directed into the indirect heat exchange clinker silo and discharged via the discharge valve. Subsequently, it undergoes cooling in the negative pressure air cooling system before storage in silos, resulting in the production of highly active MgO [117]. Liaoning Donghe New Materials Co. Ltd. extensively applies this method depicted in Fig. 7b to produce MgO [118].

### Reflection kiln calcination

In the calcination process of the reflection kiln, there are advantages such as efficient energy utilization, high thermal efficiency, simple operation, and low production costs. However, it also presents shortcomings including kiln temperature instability, high energy consumption, excessive raw material usage, inadequate product particle size control, and significant environmental pollution [119]. The reflector kiln, characterized by its simplicity and ruggedness, operates with continuous furnace operation and intermittent charging and discharging. With a history spanning 70–80 years, this technology is primarily employed for the light burning of magnesite. Typically, magnesite with a size of 100–200 mm is fed into the furnace, unsuitable for roasting powdered or crushed materials. The process yields MgO from the decomposition of magnesium carbonate and other substances [120]. Daling Tiancheng Refractory Co. Ltd., located in Dashiqiao City, Liaoning Province, has achieved industrial-scale production using this method.

### Magnesium salt direct thermal decomposition method

**Fig. 8:**
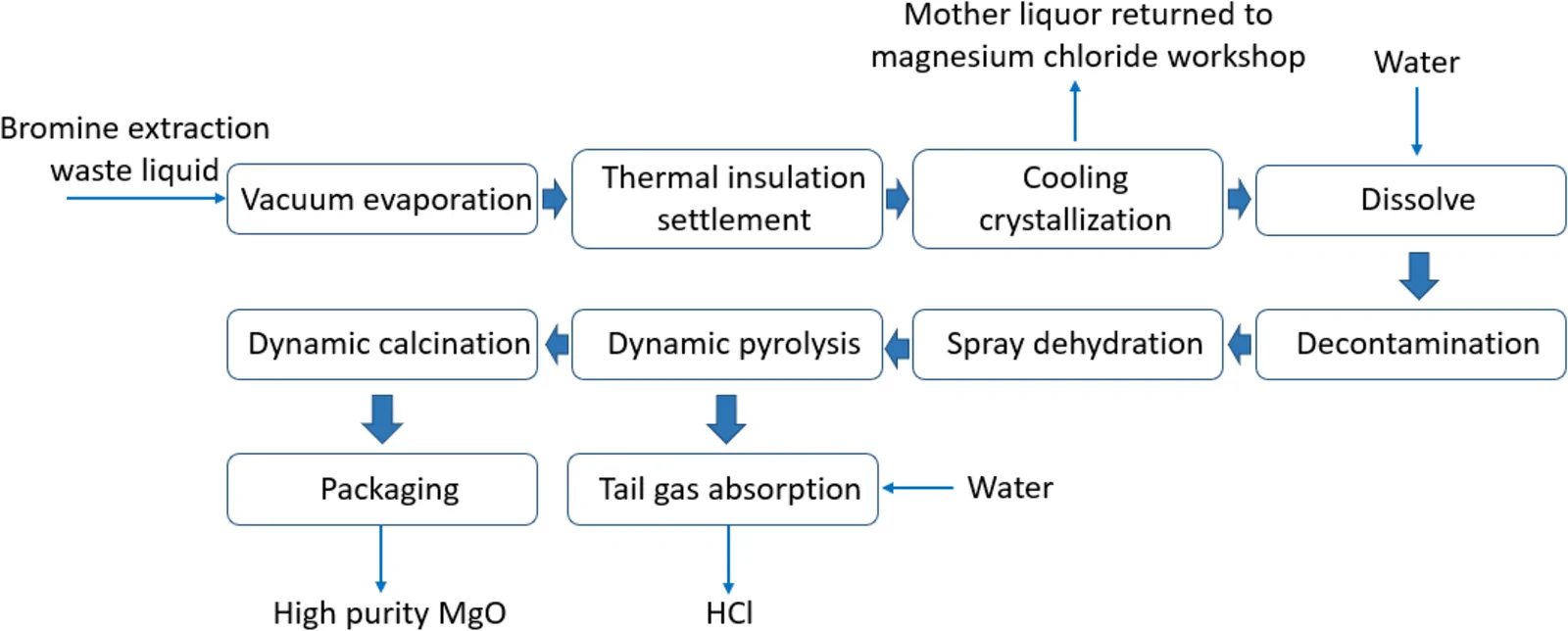
MgO production processes via magnesium salt direct thermal decomposition method

MgO can also be obtained through the thermal decomposition of aqueous magnesium chloride, achieved by heating it in either ambient air or a flow of hot air [121]. As the temperature increases, the crystallization water is gradually expelled. This process is straightforward and requires no additional auxiliary raw materials, thereby reducing production costs. Moreover, it facilitates the attainment of consistent and high-value MgO in large quantities. The spray method represents a form of direct pyrolysis, whereby brine is sprayed directly into the pyrolysis reactor for thermal decomposition, yielding crude MgO post-calcination. Following this, residual soluble chloride, which remains incompletely decomposed, is removed through multiple water washings. The crude MgO undergoes complete hydration to form Mg(OH)_2_, which is subsequently re-calcined to produce light MgO. Further calcination yields high-purity MgO, with > 99% purity. While boasting a brief pyrolysis duration and minimal production expenses, this process exhibits a drawback in its low recovery rate. The highly corrosive nature of the HCl tail gas demands sophisticated equipment, thus posing challenges in its absorption and concentration. Henan Xingfa Magnesium Industry Co. Ltd. utilizes magnesium chloride hexahydrate as a raw material to produce high-purity MgO through pyrolysis and related processes shown in Fig. 8 [122].

Shanxi Nanfeng Chemical Group utilizes high-magnesium brine from Yuncheng Salt Lake, processed through evaporation, cooling, and crystallization to obtain magnesium chloride hexahydrate, which is subsequently used as raw material for producing high-purity MgO via pyrolysis [123]. Moreover, Shandong Haihua Group employs the traditional halogen-ammonium carbonate method for the production of high-purity MgO. During the production of soda ash from raw salt using the ammonia alkali process, old halogen absorbs the calcined condensate of CO_2_. Utilizing old halogen and calcined condensate as inputs, the company employs continuous reaction pyrolysis via the ammonium carbonate method to produce high-purity MgO [124].

### Ammonia method

**Fig. 9:**
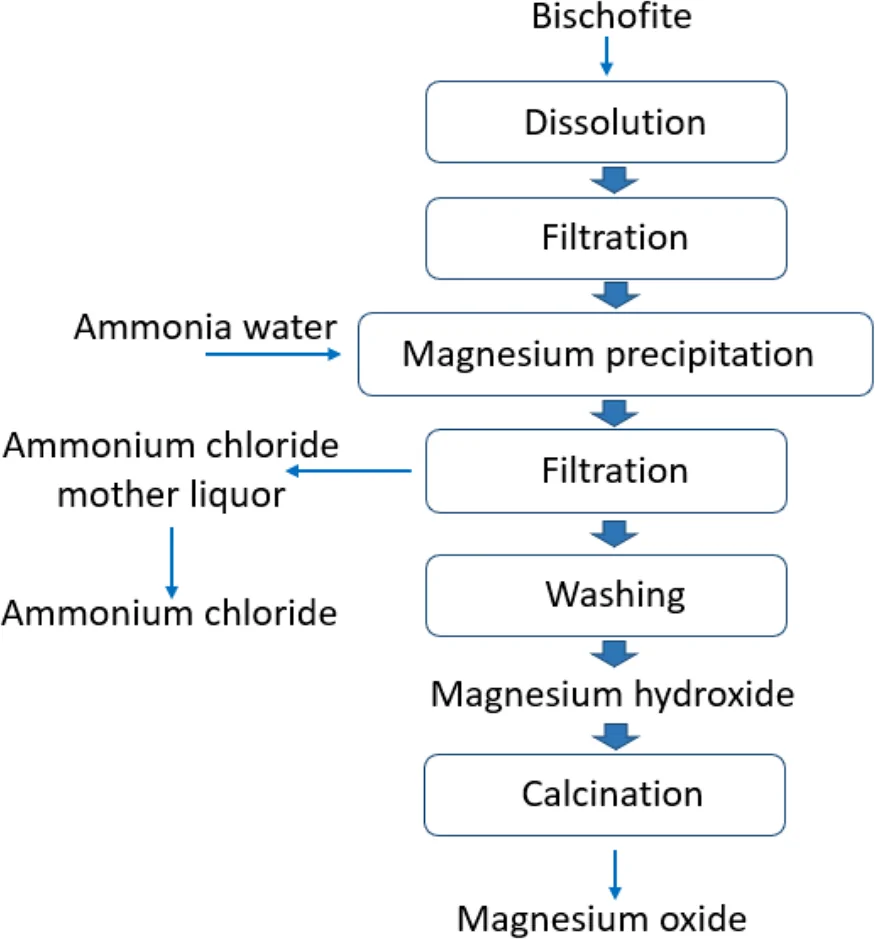
MgO production processes via ammonia method using bischofite (MgCl_2_·6H_2_O)

The ammonia process involves using ammonia or liquid ammonia as a precipitant added to an aqueous solution of magnesium chloride (or high-magnesium salt water) to precipitate magnesium hydroxide. The precipitate undergoes filtration, washing, drying, and calcination to yield MgO [125]. This method can achieve 80–85% magnesium precipitation efficiency, with an ammonia conversion rate of up to 80%, yielding a MgO mass fraction > 99% in the product. The by-product, ammonium chloride, serves as both fertilizer and chemical raw material, obviating the need for industrial waste management and minimizing environmental impact [121]. The production process flow chart is illustrated in Fig. 9. The method involving ammonia precipitation, magnesia-lime reaction, and ammonia calcination for producing high-purity magnesia has been implemented in industrial production at Qinghai West Magnesium Industry Co. Ltd.

### Brine carbonate method

**Fig. 10:**
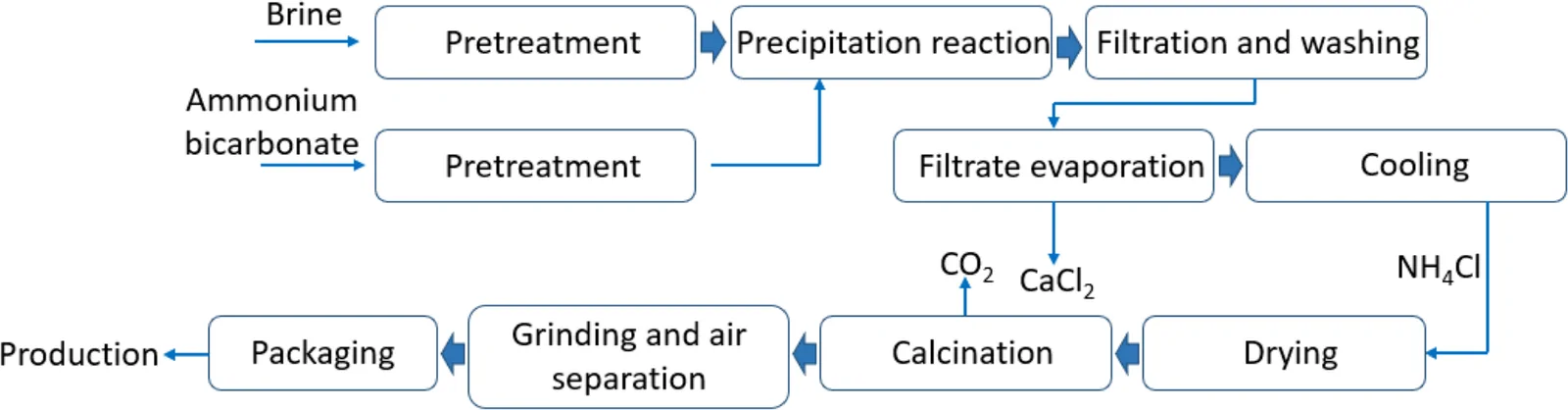
The production processes for brine carbonate method

This method entails introducing soluble carbonates into magnesium-rich brine, yielding either magnesium carbonate or basic magnesium carbonate, contingent upon the alkalinity of the carbonates. Predominantly, the carbonates incorporated consist of soda ash, ammonium carbonate, or ammonium bicarbonate [126]. To date, ammonium carbonate or ammonium bicarbonate have been utilized as precipitants [127]. Shanxi Yuncheng Baxing Chemical Co. Ltd. utilized high-magnesium brine from Yuncheng Salt Lake to produce highly active MgO using the ammonium bicarbonate method, achieving a high iodine absorption value of 140–180 mg/g [128]. The production process flow chart is illustrated in Fig. 10. This process is guided by the principles of circular economy and does not emit waste, waste gas, or residue into the environment. Its primary product is activated MgO, with NH_4_Cl as the by-product. A portion of the CO_2_ generated during the process is reclaimed for carbonation and recycling, while the remainder is captured, compressed, and cooled to produce dry ice. This dry ice finds application in the food industry and other sectors, effectively mitigating CO_2_ emissions.

## Recommendations on the characterization, categorization, handling and use of MgO

Magnesium-based cements, specifically MC and MS binders, have emerged as promising low-carbon alternatives to PC. Here, the source and processing of MgO significantly influence the performance of these cements. MgO reactivity is a critical parameter and is influenced by calcination temperature and precursor morphology. Light-burned MgO has high SSA (> 60 m^2^/g) and lower crystallite size, which supports rapid hydration and carbonation [71]. Accurate and repeatable characterization is crucial for quality assurance. A combination of methods should be employed to assess purity, thermal processing quality, and the reactivity of the produced MgO. Recommended methods include the use of BET SSA for assessing surface area and hydration reactivity, LOI for calcination efficiency and residual phases, XRF/ICP for chemical purity and contaminant detection, pH and acid neutralization tests for reactivity and buffering capacity, and XRD for phase identification and structural evolution. A combination of these methods is crucial to obtain holistic understanding and assessment about the characteristics of MgO. These outputs can enable predictive performance modelling and ensure batch-to-batch consistency of MgO-based products in large-scale and industrial production.

For handling MgO, the high reactivity of the phase can cause short shelf life due to the hydration and carbonation during storage. In the lab scale, this can be overcome by small batch size with tightly controlled storing conditions. However, in mass production, more efforts to isolate the reactive MgO from its exposure to air and humidity are necessary. Therefore, reactive MgO may require industrial airtight silos to store the material at large quantities.

The choice of MgO types and their reactivity are important steps to achieve desired performance in MgO-based cements. The properties, origin, and impurities of MgO show high probability causing the unexplained and unexpected results for MC and MS cement. Therefore, there is a need to systematically address the relationship between MgO’s characteristics and the formation and performance of these cements. For MC cements, highly reactive MgO appears to be required to enhance hydration and subsequently achieve fast carbonation of Mg(OH)2. However, MS cement seems to favor MgO of medium reactivity to avoid fast setting while still enabling good formation of M-(A-)S-H. To enhance the hydration of MgO and the workability of mixes, additives are an important factor that has been employed in both MC and MS cements for which this is the central theme in the *Working Group 4: Rheology and admixtures* of this Technical Committee. The current understanding about the characteristics of MgO also initiates a need for further research and development for suitable types of additives and admixtures for these systems and fundamental understanding about their working principles.

The long-term development for MC and MS cements is to develop standards and regulatory support to initiate guidelines on characterizing MgO reactivity, mix design parameters, and curing regimes specific to MC and MS cements. Later, the favorable characteristics of MgO will be used as input to improve and modify the industrial practices to produce, handling, and store the material. In further stages, when the technology readiness level reaches full commercial production, market integration and deployment will be targeted to move the cements from niche applications to mainstream.

## Conclusions

This review is a part of activities in the *Working Group 2Reactivity, reaction rate, and kinetics of raw materials* of RILEM Technical Committee *Magnesia-based binders in concrete* (311-MBC). Here, we provide the current understanding and practices to handle MgO i.e., the common precursors to form MgO-based cements. Key takeaways are:

- MgO may exhibit significant variation in its characteristics such as reactivity and morphology depending on the source and extraction methods. This led to the distinctive behaviors and performance in relevant MgO-based cements as seen in the open literature including some expected and unexplained behaviors compared to other types of cements. This review also provides a toolbox for different methods to evaluate the properties of MgO and suggestions of practices that can be used to handle MgO as a cement precursor.
- There are several well-established industrial processes to derive MgO and relevant technical know-how especially in China (i.e., the largest MgO producer worldwide). However, it is important to obtain comprehensive techno-economic assessment and LCA for the produced MgO to better understand the feasibility and carbon mitigation potential of cements using these MgO. This is closely linked to *Working Group 6: Road maps—feasibility and projection* in the same RILEM Technical Committee.
- The use of additives plays an important role to enhance the reactivity of MgO in relevant cements in both hydration and carbonation. There are potential breakthroughs in using additives to better control or enhance the reactivity of MgO.

## Data Availability

Data supporting the findings of this review are available within the paper.
